# Emergency Room Visits with a Non-Traumatic Dental-Related Diagnosis in Hawaii, 2016–2020

**DOI:** 10.3390/ijerph19053073

**Published:** 2022-03-05

**Authors:** Masako Matsunaga, John J. Chen, Patrick Donnelly, Carlotta Ching Ting Fok, Nancy S. Partika

**Affiliations:** 1 Department of Quantitative Health Sciences, John A. Burns School of Medicine, University of Hawai’i at Manoa, Honolulu, HI 96813, USA; jjchen@hawaii.edu; 2 Hawai’i Oral Health Coalition, Hawai’i Public Health Institute, Honolulu, HI 96813, USA; patrick@hiphi.org; 3 Hawai’i State Department of Health, Family Health Services Division, Honolulu, HI 96813, USA; chingting.fok@doh.hawaii.gov; 4 Hawaii Grassroots Oral Health Advocate-OPEN, Honolulu, HI 96816, USA; nantika@hawaii.edu

**Keywords:** emergency medical services, non-traumatic dental-related diagnosis, oral health, Medicaid, race and ethnicity, adult

## Abstract

The purpose of this study was to characterize the frequencies and patterns of emergency room (ER) visits with a non-traumatic dental-related diagnosis among adults (≥21 years old) in Hawaii, United States. This descriptive cross-sectional study used state-level inpatient and outpatient data recorded in Hawaii from 2016 to 2020. We identified dental-related ER visits using the diagnosis codes for non-traumatic dental-related conditions and summarized the frequency, rates, and cumulative total charges of the ER visits. The results show that approximately 30 thousand ER visits between 2016 and 2020 had a dental-related diagnosis. Sixty-seven percent of them had it as a principal diagnosis, amounting to USD 38.7 million total charges over the five years. A high proportion of these visits was found among those aged 21–44 years old (62%), Medicaid beneficiaries (49%), and Native Hawaiians/Part Native Hawaiians (26%). Compared to the proportions of ER visits with a secondary diagnosis, these groups had a higher proportion of ER visits with a principal diagnosis (*ps* < 0.001). A higher visit rate was found for those aged 21–44 years old and from less-populated counties. These results suggest that oral health disparities in age, socioeconomic status, and race/ethnicity exist in Hawaii. Our findings could provide insight in developing a framework to reduce oral health disparities, particularly among Medicaid beneficiaries and Native Hawaiians. Dental coverage with effective education in multiple dimensions is necessary to reduce non-traumatic dental-related ER visits.

## 1. Introduction

Oral care is essential for maintaining overall health [1]. Inadequate oral care could lead to dental-related problems, such as tooth loss and periodontal diseases, and other health issues [1,2]. Previous studies suggest that cardiovascular diseases, diabetes, kidney disease, dementia, and certain cancers are associated with oral health [3,4,5,6,7]. Moreover, an individual with a dental-related problem is more likely to have less confidence and increased stress levels [8,9]. In addition to affecting the quality of life, a lack of regular dental check-ups could lead to costly procedures, such as emergency room (ER) services [10,11,12]. According to the study that used the 2014 Nationwide Emergency Department Sample data, the average charge of dental ER visits was USD 994 for adults and USD 971 for children [10]. Therefore, the lack of regular dental check-ups could create tremendous health and financial impacts, especially on those with low incomes.

Past epidemiological studies reported that among dental-related ER visits, the proportions of young and middle-aged adults, uninsured individuals, and beneficiaries of Medicaid (US federal and state program that helps with healthcare costs for people with limited income and resources) were higher than their counterparts [10,11,12,13,14,15]. The proportion of black people was also higher than white people [13,14]. However, Asians, Native Hawaiians, and Pacific Islanders in a culturally diverse community, such as in Hawaii, have not been well-researched and reported. Unlike most other states in the United States, Hawaii has a higher proportion of these race/ethnic groups. Asians, Native Hawaiian, and Other Pacific Islanders, including multi-race individuals, account for more than a half of the Hawaii population [16]. To the best of our knowledge, no studies have been published on dental-related ER visits among the adult population in Hawaii. To fill the gap, we performed a population-based study using Hawaii hospital data. The purpose of this study was to identify and characterize all ER visits with a non-traumatic dental-related diagnosis using state-level inpatient and outpatient data in Hawaii from 2016 to 2020.

## 2. Materials and Methods

### 2.1. Data

This descriptive cross-sectional study used de-identified state-level outpatient and inpatient data obtained from the Laulima Data Alliance [17]. The data consisted of administrative records for patients who used emergency services and received any (a principal and or secondary) dental-related diagnosis in Hawaii from 2016 to 2020. Those aged 20 years and younger and those who were not Hawaii residents at the time of their ER visits were excluded, resulting in 29,536 ER visits for analysis. Each ER visit record had a visit identification, a patient-linking identification, at least one diagnosis, and the patient’s demographics.

### 2.2. Principal and Any Listed Dental-Related Diagnoses

The principal diagnoses of the medical records were identified based on the International Classification of Diseases, Tenth Revision codes (ICD-10) for non-traumatic dental conditions [18]. Supplementary Materials (Table S1) lists these ICD-10 codes. If an ER visit record had a principal (first-listed) diagnosis that matched any of these ICD-10 codes, it was defined as an ER visit with a principal dental-related diagnosis. If an ER visit record had a principal diagnosis and/or an additional diagnosis that matches any of these ICD-10 codes, it was defined as an ER visit with any listed dental-related diagnosis.

### 2.3. Other Variables

Data included the patient’s sex (male or female), age, county of residence (Honolulu, Hawaii, Maui, or Kauai), primary payment source, race/ethnicity, and total charges for each ER visit. Total charges are the dollar amounts before applying any co-pay, deductible, negotiation, or coinsurance. It may include both the insurer’s and patient’s portions of the charge. Age was categorized into four groups (21–44, 45–64, 65–84, or +85 years old). The primary payment source was classified into five groups (Private, Medicaid, Medicare, Self-Pay, or Other) [19]. Medicaid and Medicare are US federal and state programs to help with healthcare costs for eligible individuals. People with limited income and resources are eligible for Medicaid. Medicare is available for those aged 65 or older, younger individuals with disabilities, and individuals with end-stage renal disease. Race/ethnicity was categorized into seven groups (White, Native Hawaiian/Part Native Hawaiian, Pacific Islander, Filipino, Japanese, Other Asian, or Other Race) [20,21]. The Other Race category included Hispanic, Native American, Black, and other races.

### 2.4. Data Analysis

We performed descriptive analyses of ER visits with a principal and any listed non-traumatic dental-related diagnosis. We obtained the frequencies and proportions of ER visits by sociodemographic characteristics (sex, age groups, county of residence, primary payment source, and race/ethnicity) for each year (2016–2020). A small number of ER visits had a missing value of the primary payment source (*n* = 14, 0.04%) or race/ethnicity (*n* = 433, 1.2%). The primary payment source and race/ethnicity percentages were obtained after excluding ER visits with a missing value. We summarized the frequency and proportion of patients with multiple ER visits and the cumulative total charges before and after excluding ER visits resulting in an inpatient admission. We performed chi-square goodness-of-fit tests to examine whether there was no difference between the observed and expected frequencies of the ER visits with a dental-related diagnosis over the five years (expected percentage for each year: 100%/5 years = 20.0%); chi-square tests of independence to estimate the relationships between an ER visit with a dental-related diagnosis (principal or secondary only) and a sociodemographic factor (sex, age, primary payment source, and race/ethnicity) in the United States. A *p* value less than 0.05 was considered statistically significant. The rate of ER visits with a dental-related diagnosis per 100,000 persons was obtained for each calendar year by age groups and by county of residence. The Hawaii population estimates were derived from the American Community Survey [16]. Because estimates for 2020 are not available at the time of the analysis, we only calculated the rates for 2016 to 2019. All analyses were conducted by R Statistical software (version 4.0.2).

## 3. Results

### 3.1. Frequency of ER Visits with a Non-Traumatic Dental-Related Diagnosis

Table 1 shows the frequencies of ER visits with a non-traumatic dental-related diagnosis recorded for adult patients in Hawaii. From 2016 to 2020, a total of 29,536 visits were recorded with any listed dental-related diagnoses. The number of ER visits from 2016 to 2019 appears stable but decreased in 2020 compared to the prior four years (*p* < 0.001). Sixty-seven percent of the ER visits had a principal dental-related diagnosis (*n* = 19,691). The percentages were similar across the years (2016–2020: 66%, 67%, 67%, 68%, 65%). However, the number of ER visits with a principal dental-related diagnosis decreased in 2020 (*p* < 0.001). Among the ER visits with a principal dental-related diagnosis, a high proportion was found for those aged 21–44 years old (62%) and those living in Honolulu County (57%). A high proportion was also found for those who used Medicaid as a primary payment source (Medicaid beneficiaries) (49%), white people (30%), and Native Hawaiians/Part Native Hawaiians (26%). These sociodemographic characteristics were consistent across the years. About 16% of the patients visited more than once over the five years, and 10–11% visited more than once each year.

The results of chi-square tests show that the age group, primary payment source, and race/ethnicity were associated with an ER visit with a principal dental-related diagnosis (*p* < 0.001 for all). Compared to the proportion of ER visits with a secondary dental-related diagnosis, a higher proportion was found for those aged 21–44 years old, Medicaid beneficiaries, and Native Hawaiians/Part Native Hawaiians. Supplementary Materials (Table S3) presents detailed results.

### 3.2. ER Visits by Race/Ethnicity

**Figure 1:**
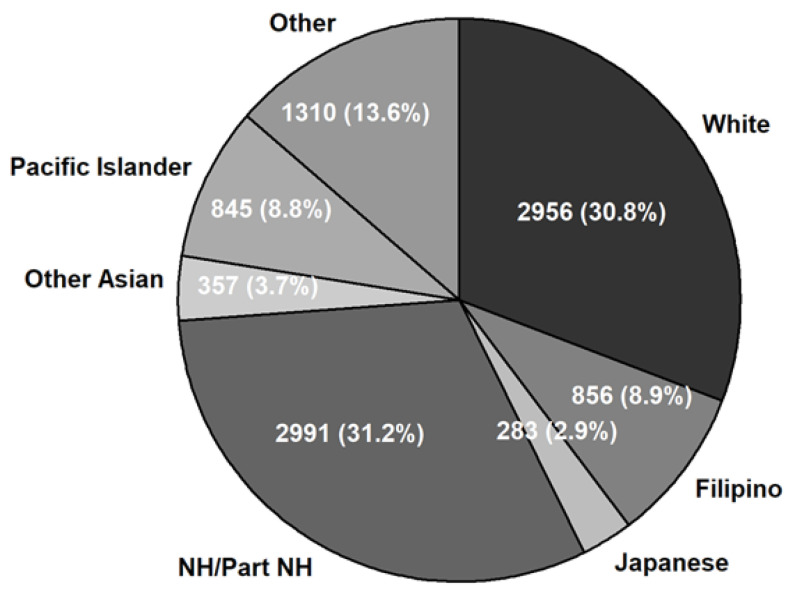
Emergency room visits with a principal non-traumatic dental-related diagnosis by race and ethnicity among Medicaid beneficiaries, Hawaii 2016–2020 (*n* = 9680). NH: Native Hawaiian.

Figure 1 shows the frequencies and percentages of ER visits with a principal dental-related diagnosis by race/ethnicity groups among Medicaid beneficiaries. The proportions of white people and Native Hawaiians/Part Native Hawaiians on Medicaid were higher, at 31% each.

### 3.3. Cumulative Total Charges

The cumulative total charges of ER visits with a principal dental-related diagnosis over the five years was USD 38.7 million (Table 2). After excluding the ER visits resulting in an inpatient admission, the cumulative total charges were USD 27.5 million. Nearly half of the ER visits were for patients who used Medicaid as a primary payment source (45%). Private health insurance companies (30%) and Medicare (16%) were most common after Medicaid. This pattern was observed throughout the five years. The results of the ER visits with any listed dental-related diagnosis show the same patterns for demographic characteristics (Table S2).

### 3.4. Rates of ER Visits with a Principal Non-Traumatic Dental-Related Diagnosis

**Figure 2:**
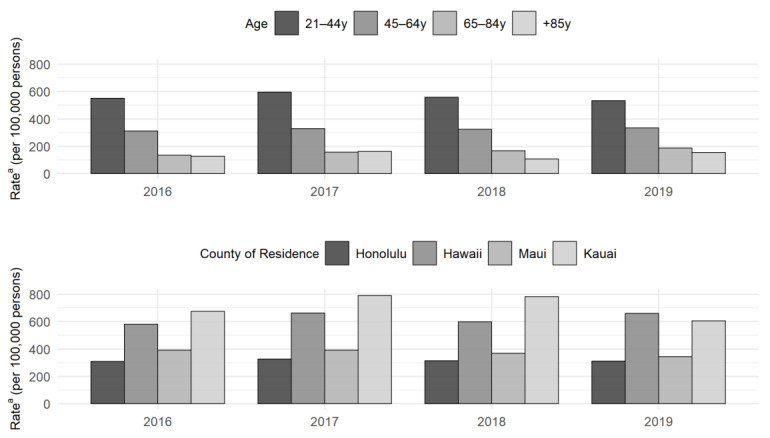
Rates ^a^ of emergency room visits with a principal non-traumatic dental-related diagnosis (per 100,000 persons) by age group and county of residence for adults aged 21 years and older in Hawaii 2016–2019. ^a^ The rate was calculated using the number of emergency room visits with a principal non-traumatic dental-related diagnosis in the numerator and the Hawaii population estimate in the denominator. The Hawaii population estimate was derived from the American Community Survey [16].

The rate of ER visits with a principal dental-related diagnosis was nearly 400 per 100,000 persons each year (2016–2019: 375, 404, 384, 380). Figure 2 shows the rates by age group and county of residence. The 21–44-year-old group had a higher rate (2016–2019: 550, 594, 559, 532), followed by the 45–64-year-old group. Despite the lower frequency, the visit rates for less-populated counties (Hawaii and Kuai) were higher than those of the more populated counties (2016–2019: Honolulu, 309, 326, 313, 311; Hawaii, 579, 663, 597, 661; Maui, 392, 392, 370, 344; Kauai, 676 788, 783, 605). Detailed results of the rates can be found in Supplementary Materials (Table S4).

## 4. Discussion

Our descriptive cross-sectional study found that nearly 20,000 ER visits were recorded with a principal non-traumatic dental-related diagnosis in Hawaii from 2016 to 2020. Although the number slightly decreased in 2020, likely due to restrictions imposed by the COVID-19 pandemic, it consistently reached more than four thousand visits each year from 2016 to 2020. Our findings show a high proportion of Medicaid beneficiaries and higher rates of adults aged 21 to 44 years old and those living in rural areas utilizing the ER for acute non-traumatic dental-related conditions. The results of statistical analyses also support that the utilization of ERs for non-traumatic dental-related conditions was associated with age group and primary payment source. These findings are consistent with previous studies using the national data [10,11,12,14,15].

To the authors’ best knowledge, this is the first published study examining the racial/ethnic characteristics of non-traumatic dental-related ER visits in Hawaii. Compared to most states in the United States, Hawaii is a more culturally diverse state. In addition, the proportions of Asians, Native Hawaiians, and other Pacific Islanders are higher than in the other states. Based on the 2019 American Community Survey [16], Native Hawaiians/Part Native Hawaiians (Native Hawaiian alone or in any combination) account for 16.5% among Hawaii population aged 21 years and older. This study found a higher proportion among this race group (26%) utilizing ERs. Among the ER visits by Medicaid beneficiaries, the proportion of Native Hawaiians/Part Native Hawaiians was even higher (31%). On the other hand, the proportions of Filipino (11%) and Japanese (5%) in the dental-related ER visits appears to have been lower than the corresponding Hawaii population size (17% for Filipino alone and 15% for Japanese alone in 21 years and older). Thus, our findings suggest that oral health disparities exist for Native Hawaiians/Part Native Hawaiians due to a lack of access to affordable, preventive oral health services.

Acute oral care in emergency rooms is limited to temporary and palliative care, delaying appropriate definitive dental care [12,22,23]. Numerous studies discussed dental-related ER visits concerning systematic health issues consequently occur by poor dental status and tremendous medical costs to both the patient and the healthcare system [3,5,6,10,11,12,13,14,23,24]. Our study found that Hawaii also had high total charges for dental-related ER visits. As one of the Healthy People 2030 objectives is to increase the proportion of people with dental insurance, dental coverage is crucial for maintaining overall health. Past studies have reported a high proportion of uninsured individuals’ ER utilization for non-traumatic dental-related conditions. Our findings show that most patients had health insurance, likely due to the existing state law (the Hawaii Prepaid Health Care Act). This law requires private employers to provide health insurance for employees who work at least 20 h per week consecutively for four weeks [25]. In our study, more than a quarter of the ER visits were by individuals with private medical insurance. However, the data did not indicate if their insurance had dental coverage. Additionally, Hawaii is one of the few states that provide emergency-only oral health dental coverage to Medicaid beneficiaries aged 21 years and older [26]. Our findings could be informative in the discussion of encouraging dental coverage to all individuals and expanding Medicaid preventive and restorative dental coverage for the adult population. According to previous studies conducted in the United States, those with low income and a low education level are more likely to have poor oral health and use ER services for non-traumatic dental conditions [11,12,27]. Given the associations, effective education programs addressing the importance of oral care and regular check-ups are necessary for this community. It may ultimately help improve the overall health of vulnerable people and prevent unnecessary medical expenses.

Other limitations of the current study include potential misclassification of ER visits and their demographic characteristics. The analysis did not include acute dental-related visits in non-ER settings, such as urgent care clinics. Our data did not include ER visits without any listed non-traumatic dental-related diagnosis. Thus, we could not explore significant factors contributing to ER visits for non-traumatic dental-related conditions by comparing them with ER visits for other conditions. Despite these limitations, the current study comprehensively described ER visits with a non-traumatic dental-related diagnosis using population data in Hawaii. Another strength is that our data included a high proportion of Asians, Native Hawaiians, and Pacific Islanders, which is unique to the state of Hawaii. Thus, the results could provide valuable insights regarding these race groups. Our findings suggest that significant health disparities in oral health exist among the adult population in Hawaii. Further research is needed to find effective approaches to eliminate these disparities and reduce the overall number of ER visits for non-traumatic dental-related conditions.

## 5. Conclusions

Using population non-traumatic dental ER data, our study revealed oral health disparities related to age, race/ethnicity, and socioeconomic status in Hawaii. Our findings support future policy analysis and systemic changes to help those at-risk improve their oral and overall health and reduce high-cost healthcare. Further studies are needed to search for practical approaches to reduce the number of ER visits for non-traumatic dental-related conditions.

## Figures and Tables

**Table 1:** Descriptive analysis of emergency room (ER) visits with a non-traumatic dental-related (NTDR) diagnosis for adults aged 21 years and older in Hawaii 2016–2020.

	Total	2016	2017	2018	2019	2020
	(2016–2020)					
ER visits with any listed NTDR diagnosis, *n* (%) ^a^	29,536	6051 (20.5)	6475 (21.9)	6114 (20.7)	5992 (20.3)	4904 (16.6)
ER visits with a principal NTDR diagnosis, *n* (%) ^b^	19,691 (66.7)	4009 (66.3)	4345 (67.1)	4103 (67.1)	4063 (67.8)	3171 (64.7)
Sex, *n* (%) ^c^						
Male	10,129 (51.4) ^d,^†	1990 (49.6)	2237 (51.5)	2083 (50.8)	2120 (52.2)	1699 (53.6)
Female	9562 (48.6)	2019 (50.4)	2108 (48.5)	2020 (49.2)	1943 (47.8)	1472 (46.4)
Age, *n* (%) ^c^						
21–44 y	12,138 (61.6) ^d,^***	2554 (63.7)	2770 (63.8)	2551 (62.2)	2415 (59.4)	1848 (58.3)
45–64 y	5514 (28.0)	1129 (28.2)	1172 (27.0)	1139 (27.8)	1158 (28.5)	916 (28.9)
65–84 y	1782 (9.0)	277 (6.9)	344 (7.9)	370 (9.0)	432 (10.6)	359 (11.3)
+85 y	257 (1.3)	49 (1.2)	59 (1.4)	43 (1.0)	58 (1.4)	48 (1.5)
County of Residence, *n* (%) ^c^						
Honolulu	11,118 (56.5)	2297 (57.3)	2428 (55.9)	2311 (56.3)	2292 (56.4)	1790 (56.4)
Hawaii	4515 (22.9)	869 (21.7)	1004 (23.1)	903 (22.0)	1005 (24.7)	734 (23.1)
Maui	2248 (11.4)	481 (12.0)	490 (11.3)	467 (11.4)	438 (10.8)	372 (11.7)
Kauai	1810 (9.2)	362 (9.0)	423 (9.7)	422 (10.3)	328 (8.1)	275 (8.7)
Primary Payment Source, *n* (%) ^c^						
Medicaid	9680 (49.2) ^d,^***	2066 (51.6)	2175 (50.1)	2033 (49.6)	1826 (45.0)	1580 (49.9)
Private	5481 (27.8)	1079 (26.9)	1222 (28.1)	1160 (28.3)	1157 (28.5)	863 (27.2)
Medicare	2587 (13.1)	497 (12.4)	554 (12.8)	503 (12.3)	612 (15.1)	421 (13.3)
Self-Pay	1406 (7.1)	242 (6.0)	286 (6.6)	305 (7.4)	343 (8.4)	230 (7.3)
Other	528 (2.7)	123 (3.1)	108 (2.5)	101 (2.5)	122 (3.0)	74 (2.3)
Race/ethnicity, *n* (%) ^c^						
White	5738 (29.5) ^d,^***	1220 (30.7)	1256 (29.2)	1164 (28.7)	1185 (29.5)	913 (29.1)
Native Hawaiian (NH)/Part NH	5018 (25.8)	1035 (26.1)	1144 (26.6)	988 (24.3)	1025 (25.5)	826 (26.3)
Pacific Islander	2182 (11.2)	418 (10.5)	510 (11.8)	461 (11.4)	450 (11.2)	343 (10.9)
Filipino	2234 (11.5)	463 (11.7)	465 (10.8)	512 (12.6)	442 (11.0)	352 (11.2)
Japanese	1018 (5.2)	211 (5.3)	239 (5.6)	210 (5.2)	198 (4.9)	160 (5.1)
Other Asian	865 (4.4)	146 (3.7)	171 (4.0)	189 (4.7)	195 (4.9)	164 (5.2)
Other race	2427 (12.5)	476 (12.0)	521 (12.1)	534 (13.2)	518 (12.9)	378 (12.1)
Number of Patients, *n* (%) ^e^						
Patients with one visit	13,204 (84.3)	3091 (89.3)	3393 (89.8)	3,192 (89.2)	3273 (90.8)	2513 (90.2)
Patients with >1 visits	2465 (15.7)	372 (10.7)	387 (10.2)	388 (10.8)	331 (9.2)	274 (9.8)

^a^ Percentage of ER visits with any listed NTDR diagnosis from 2016 to 2020. ^b^ Percentage of ER visits with any listed NTDR diagnosis. ^c^ Percentage of ER visits with a principal NTDR diagnosis. ^d^ A chi-square test of independence to estimate the relationships between an ER visit with a dental-related diagnosis (principal or secondary only) and a sociodemographic factor in the United States. † *p* ≥ 0.05, *** *p* < 0.001. Supplementary Materials (Table S2) presents the frequencies of ER visits with a secondary NTDR (any listed—principal = secondary) and detailed results. ^e^ Percentage of patients who used an ER and received a principal NTDR diagnosis.

**Table 2:** Cumulative total charges ^a^ of emergency room visits with a principal non-traumatic dental-related diagnosis for adults aged 21 years and older in Hawaii 2016–2020.

	Total	2016	2017	2018	2019	2020
	(2016–2020)					
Cumulative Total Charges ^a^, million	38.7	6.6	7.3	8.1	9.9	6.7
Cumulative Total Charges ^a^, million ^b^	27.5	4.9	5.7	5.7	6.2	5.1
Cumulative Total Charges ^a^ by Patient Type with Primary Payment Source, million ^b^ (%)						
Medicaid	12.3 (44.8)	2.4 (47.8)	2.6 (46.2)	2.6 (45.2)	2.5 (40.9)	2.3 (44.7)
Private	8.1 (29.6)	1.4 (28.5)	1.6 (29.2)	0.9 (15.3)	1.8 (29.8)	1.6 (30.7)
Medicare	4.5 (16.3)	0.8 (15.5)	0.9 (16.2)	1.7 (29.6)	1.1 (18.4)	0.8 (15.9)
Self-pay	1.8 (6.5)	0.3 (5.1)	0.3 (5.7)	0.4 (7.2)	0.5 (7.7)	0.3 (6.7)
Other	0.7 (2.7)	0.2 (3.0)	0.2 (2.8)	0.1 (2.6)	0.2 (3.1)	0.1 (2.0)

^a^ A total charge is the dollar amount the hospital billed for a visit with a principal non-traumatic dental-related diagnosis. The charged amount was not usually paid in full and was necessarily paid solely by the primary insurer. ^b^ Cumulative total charges after excluding emergency room visits resulting in an inpatient admission.

## Data Availability

The data used for the current analysis is available from the Laulima Data Alliance.

## Supplementary Materials

The following supporting information can be downloaded at: https://www.mdpi.com/article/10.3390/ijerph19053073/s1, Table S1: The International Classification of Diseases, Tenth Revision (ICD-10) codes for non-traumatic dental-related diagnoses; Table S2: Descriptive analysis of emergency room visits with any listed dental-related diagnosis for adults aged 21 years and older in Hawaii 2016–2020; Table S3: Chi-square tests of independence for non-traumatic dental-related diagnoses and demographic factors among emergency room (ER) visits with any listed non-traumatic dental-related diagnosis, adults aged 21 years and older in Hawaii 2016–2020. Table S4: Rates of emergency room visits with a non-traumatic dental-related diagnosis, adults aged 21 years and older in Hawaii 2016–2019.
