# Supplementary material for: Emergency Room Visits with a Non-Traumatic Dental-Related Diagnosis in Hawaii, 2016–2020
Source: Int J Environ Res Public Health. 2022 Mar 5;19(5):3073. doi: 10.3390/ijerph19053073 (PMC8910124; doi:10.3390/ijerph19053073)
Supplement: Supplementary file 1 [file ijerph-19-03073-s001.zip › ijerph-1596883-supplementary/Table S1.pdf]

Table S1. The International Classification of Diseases, Tenth Revision (ICD-10) codes for non-traumatic dental-related diagnoses [18]

A690, A691, K00, K000, K001, K002, K003, K004, K005, K006, K007, K008, K009, K01, K010, K011, K02, K023, K025, K0251, K0252, K0253, K026, K0261, K0262, K0262, K0263, K027, K029, K03, K030, K031, K032, K033, K034, K035, K036, K037, K038, K0381, K0389, K039, K04, K040, K0401, K0402, K041, K042, K043, K044, K045, K046, K047, K048, K049, K0490, K0499, K05, K050, K0500, K0501, K051, K0510, K0511, K052, K0520, K0521, K05211, K05212, K05213, K05219, K0522, K05221, K05222, K05223, K05229, K053, K0530, K0531, K05311, K05312, K05313, K05319, K0532, K05321, K05322, K05323, K05329, K054, K0540, K055, K056, K06, K060, K0601, K06010, K06011, K06012, K06013, K0602, K06020, K06021, K06022, K06023, K061, K063, K068, K069, K08, K080, K081, K0810, K08101, K08102, K08103, K08104, K08109, K0812, K08121, K08122, K08123, K08124, K08129, K0813, K08131, K08132, K08133, K08134, K08139, K0819, K08191, K08192, K08193, K08194, K08199, K082, K0820, K0821, K0822, K0823, K0824, K0825, K0826, K083, K084, K0840, K08401, K08402, K08403, K08404, K08409, K0842, K08421, K08422, K08423, K08424, K08429, K0843, K08431, K08432, K08433, K08434, K08439, K0849, K08491, K08492, K08493, K08494, K08499, K0850, K0851, K0852, K0853, K08530, K08531, K08539, K0854, K0855, K0856, K0859, K088, K0889, K089, K09, K090, K091, K098, K099, K11, K110, K111, K112, K1120, K1121, K1122, K1123, K113, K114, K115, K116, K117, K118, K119, K12, K120, K121, K122, K123, K1230, K1231, K1232, K1233, K1239, K13, K130, K131, K132, K1321, K1322, K1323, K1324, K1329, K133, K134, K135, K136, K137, K1370, K1379, K14, K140, K141, K142, K143, K144, K145, K146, K148, K149, M26, M260, M2600, M2601, M2602, M2603, M2604, M2605, M2606, M2607, M2609, M261, M2610, M2611, M2612, M2619, M262, M2620, M2621, M26211, M26212, M26213, M26219, M2622, M26220, M26221, M2623, M2624, M2625, M2629, M263, M2630, M2631, M2632, M2633, M2634, M2635, M2636, M2637, M2639, M264, M265, M2650, M2651, M2652, M2653, M2654, M2655, M2656, M2657, M2659, M266, M2660, M26601, M26602, M26603, M26609, M2661, M26611, M26612, M26613, M26619, M2662, M26621, M26622, M26623, M26629, M2663, M26631, M26632, M26633, M26639, M2669, M267, M2670, M2671, M2672, M2673, M2674, M2679, M268, M2681, M2682, M2689, M269, M27, M271, M272, M273, M274, M2740, M2749, M275, M2751, M2752, M2753, M2759, M276, M2761, M2762, M2763, M2769, M278, M279, M7911, R682, R6884, Z012, Z0120, Z0121, Z463, Z464
