# Supplementary material for: Emergency Room Visits with a Non-Traumatic Dental-Related Diagnosis in Hawaii, 2016–2020
Source: Int J Environ Res Public Health. 2022 Mar 5;19(5):3073. doi: 10.3390/ijerph19053073 (PMC8910124; doi:10.3390/ijerph19053073)
Supplement: Supplementary file 1 [file ijerph-19-03073-s001.zip › ijerph-1596883-supplementary/Table S2.pdf]

Table S2. Descriptive analysis of emergency room (ER) visits with any listed non-traumatic dental-related (NTDR) diagnosis: adults aged 21 years and older in Hawaii 2016–2020

	Total	Year				
	(2016-2020)	2016	2017	2018	2019	2020
Emergency room visits with any listed diagnosis, *n* (%)[a]	29,536	6051 (20.5)	6475 (21.9)	6114 (20.3)	5992 (20.3)	4904 (16.6)
Sex, *n* (%)[b]						
Male	15,089 (51.1)	1990 (49.6)	2237 (51.5)	2083 (50.8)	2120 (52.2)	1699 (53.6)
Female	14,447 (48.9)	2019 (50.4)	2108 (48.5)	2020 (49.2)	1943 (47.8)	1472 (46.4)
Age, *n* (%)[b]						
21–44 y	16,497 (55.9)	3490 (57.7)	3800 (58.7)	3457 (56.5)	3222 (53.8)	2528 (51.5)
45–64 y	8599 (29.1)	1785 (29.5)	1786 (27.6)	1765 (28.9)	1761 (29.4)	1502 (30.6)
65–84 y	3704 (12.5)	639 (10.6)	719 (11.1)	750 (12.3)	868 (14.5)	728 (14.8)
+85 y	736 (2.5)	137 (2.3)	170 (2.6)	142 (2.3)	141 (2.4)	146 (3.0)
County of Residence, *n* (%)[b]						
Honolulu	18,126 (61.4)	3728 (61.6)	3930 (60.7)	3709 (60.7)	3679 (61.4)	3080 (62.8)
Hawaii Island	6015 (20.4)	1179 (19.5)	1332 (20.6)	1250 (20.4)	1282 (21.4)	972 (19.8)
Maui	2961 (10.0)	627 (10.4)	646 (10.0)	614 (10.0)	587 (9.8)	487 (9.9)
Kauai	2434 (8.2)	517 (8.5)	567 (8.8)	541 (8.8)	444 (7.4)	365 (7.4)
Primary Payment Source, *n* (%)[b]						
Medicaid	13,473 (45.6)	2922 (48.3)	3022 (46.7)	2786 (45.6)	2503 (41.8)	2240 (45.7)
Private	8065 (27.3)	1619 (26.8)	1775 (27.4)	1714 (28.0)	1647 (27.5)	1310 (26.7)
Medicare	5200 (17.6)	1007 (16.6)	1109 (17.1)	1014 (16.6)	1167 (19.5)	903 (18.4)
Self-pay	1923 (6.5)	312 (5.2)	388 (6.0)	423 (6.9)	482 (8.1)	318 (6.5)
Other	861 (2.9)	189 (3.1)	181 (2.8)	175 (2.9)	188 (3.1)	128 (2.6)
Race/Ethnicity, *n* (%)[b]						
White	8608 (29.4)	1828 (30.5)	1878 (29.2)	1762 (29.1)	1740 (29.4)	1400 (28.8)
Native Hawaiian (NH)/Part NH	6897 (23.6)	1431 (23.8)	1557 (24.2)	1371 (22.7)	1379 (23.3)	1159 (23.9)
Pacific Islander	3177 (10.9)	617 (10.3)	728 (11.3)	657 (10.9)	670 (11.3)	505 (10.4)
Filipino	3586 (12.3)	742 (12.4)	758 (11.8)	773 (12.8)	696 (11.7)	617 (12.7)
Japanese	1994 (6.8)	424 (7.1)	440 (6.9)	384 (6.4)	402 (6.8)	344 (7.1)
Other Asian	1542 (5.3)	289 (4.8)	307 (4.8)	323 (5.3)	328 (5.5)	295 (6.1)
Other race	3448 (11.8)	669 (11.2)	754 (11.7)	775 (12.8)	712 (12.0)	538 (11.1)
Number of Patients, *n* (%)[c]						
Patients with one visit	19,299 (83.4)	4569 (88.5)	4983 (89.4)	4727 (88.9)	4696 (89.9)	3834 (89.7)
Patients with >1 visits	3839 (16.6)	594 (11.5)	592 (10.6)	590 (11.1)	438 (10.1)	342 (10.3)
Cummulative Total Charges, M[d]	276.4	51.4	50.5	51.0	57.6	65.8
Cummulative Total Charges, M[e]	54.7	9.7	11.2	11.2	12.1	10.6

Cummulative Total Charges by Patient Type wih Primary Payment Source, M[e] (%)						
Medicaid	21.7 (39.7)	4.1 (42.4)	4.4 (39.8)	4.3 (39.0)	4.5 (37.2)	4.4 (40.9)
Private	16.1 (29.4)	3.0 (31.3)	3.2 (28.8)	3.4 (30.8)	3.3 (27.5)	3.1 (29.1)
Medicare	11.5 (21.1)	1.9 (19.1)	2.5 (22.8)	2.2 (19.8)	2.8 (22.8)	2.2 (20.5)
Self-pay	3.3 (6.1)	0.4 (4.0)	0.6 (5.4)	0.7 (6.7)	1.0 (8.0)	0.6 (5.9)
Other	2.0 (3.7)	0.3 (3.3)	0.4 (3.2)	0.4 (3.7)	0.5 (4.5)	0.4 (3.5)

[a] Percentage of ER visits with any listed NTDR diagnosis from 2016 to 2020. [b] Percentage of ER visits with any listed NTDR diagnosis. [c] Percentage of patients who used an ER and received any listed NTDR diagnosis. [d] A total charge is the dollar amount the hospital billed for an ER visit with any listed NTDR diagnosis. [e] Cumulative total charges after excluding ER visits resulted in an inpatient admission.
