# Supplementary material for: Emergency Room Visits with a Non-Traumatic Dental-Related Diagnosis in Hawaii, 2016–2020
Source: Int J Environ Res Public Health. 2022 Mar 5;19(5):3073. doi: 10.3390/ijerph19053073 (PMC8910124; doi:10.3390/ijerph19053073)
Supplement: Supplementary file 1 [file ijerph-19-03073-s001.zip › ijerph-1596883-supplementary/Table S3.pdf]

Table S3. Chi-square tests of independence for non-traumatic dental-related diagnoses and demographic factors among emergency room (ER) visits with any listed dental-related non-traumatic diagnosis: adults aged 21 years and older in Hawaii 2016–2020

	Total: ER visits with any listed non-traumatic dental-related diagnosis (n = 29,536)	ER visits with a principal non-traumatic dental-related diagnosis (n = 19,691)	ER visits with a secondary non-traumatic dental-related diagnosis (n = 9845)	*P*-value[a]
Sex, *n* (%)				
Male	15,089 (51.1)	10,129 (51.4)	4960 (50.4)	0.086
Female	14,447 (48.9)	9562 (48.6)	4885 (49.6)	
Age, *n* (%)				
21–44 y	16,497 (55.9)	12,138 (61.6)	4359 (44.3)	<0.001
45–64 y	8599 (29.1)	5514 (28.0)	3085 (31.3)	
65–84 y	3704 (12.5)	1782 (9.0)	1922 (19.5)	
+85y	736 (2.5)	257 (1.3)	449 (4.9)	
Primary Payment Source, *n* (%)[b]				
Medicaid	13,473 (45.6)	9680 (49.2)	2613 (26.6)	<0.001
Private	8065 (27.3)	5481 (27.8)	3793 (38.5)	
Medicare	5200 (17.6)	2587 (13.1)	2584 (26.3)	
Self-pay	1923 (6.5)	1406 (7.1)	517 (5.3)	
Other	861 (2.9)	528 (2.7)	333 (3.4)	
Race/Ethnicity, *n* (%)[b]				
White	8608 (29.4)	5738 (29.5)	2870 (29.4)	<0.001
Native Hawaiian (NH)/Part NH	6897 (23.6)	5018 (25.8)	1879 (19.2)	
Pacific Islander	3177 (10.9)	2182 (11.2)	995 (10.2)	
Filipino	3586 (12.3)	2234 (11.5)	1352 (13.8)	
Japanese	1994 (6.8)	1018 (5.2)	976 (10.0)	
Other Asian	1542 (5.3)	865 (4.4)	677 (6.9)	
Other race	3448 (11.8)	2427 (12.5)	1021 (10.5)	

[a] *P*-values were obtained from chi-squared tests of independence to examine the relationships between non-traumatic dental-related diagnoses (principal or secondary only) and sociodemographic factors in the US. [b] Percentage was obtained after excluding emergency room visits with a missing value of the variable (Primary Payment Source: *n*=14, 0.04%; Race/ethnicity: *n*=433, 1.2%).
