# Supplementary material for: Emergency Room Visits with a Non-Traumatic Dental-Related Diagnosis in Hawaii, 2016–2020
Source: Int J Environ Res Public Health. 2022 Mar 5;19(5):3073. doi: 10.3390/ijerph19053073 (PMC8910124; doi:10.3390/ijerph19053073)
Supplement: Supplementary file 1 [file ijerph-19-03073-s001.zip › ijerph-1596883-supplementary/Table S4.pdf]

Table S4. Rates[a] of emergency room visits with a non-traumatic dental-related diagnosis: Adults aged 21 years and older in Hawaii 2016–2020

	Principal Diagnosis				Any Listed Diagnosis			
Rate[a] (vists/100,000 persons)	2016	2017	2018	2019	2016	2017	2018	2019
Total	374.6	403.8	383.7	379.8	565.4	601.7	571.7	560.1
Age								
21–44 y	549.8	594.1	558.9	531.9	751.3	815.0	757.4	709.6
45–64 y	311.7	329.1	324.0	334.4	492.8	501.6	502.1	508.5
65–84 y	135.0	158.4	167.1	186.4	311.5	331.0	338.8	374.5
+85 y	127.5	161.5	107.3	154.0	356.5	465.4	354.4	374.3
County of Residence								
Honolulu	308.9	325.5	313.2	311.3	501.3	526.9	502.7	499.7
Hawaii	579.2	663.2	596.7	661.2	785.8	879.9	826.0	843.4
Maui	391.6	391.5	369.8	344.2	510.4	516.1	486.2	461.3
Kauai	675.5	788.2	782.7	604.5	964.8	1056.5	1003.5	818.3

[a] A rate is calculated using the number of emergency room visits with a principal non-traumatic dental-related diagnosis in the numerator and the Hawaii population estimate in the denominator. The Hawaii population estimate was derived from the American Community Survey [16].
